# Biocatalytic Approaches to Building Blocks for Enzymatic and Chemical Glycan Synthesis

**DOI:** 10.1021/jacsau.2c00529

**Published:** 2022-12-07

**Authors:** Jonathan P. Dolan, Sebastian C. Cosgrove, Gavin J. Miller

**Affiliations:** School of Chemical and Physical Sciences & Centre for Glycosciences, Keele University, Keele, Staffordshire ST5 5BG, United Kingdom

**Keywords:** biocatalysis, chemoenzymatic synthesis, enzymes, glycan, sugar nucleotide, glycobiology, carbohydrate

## Abstract

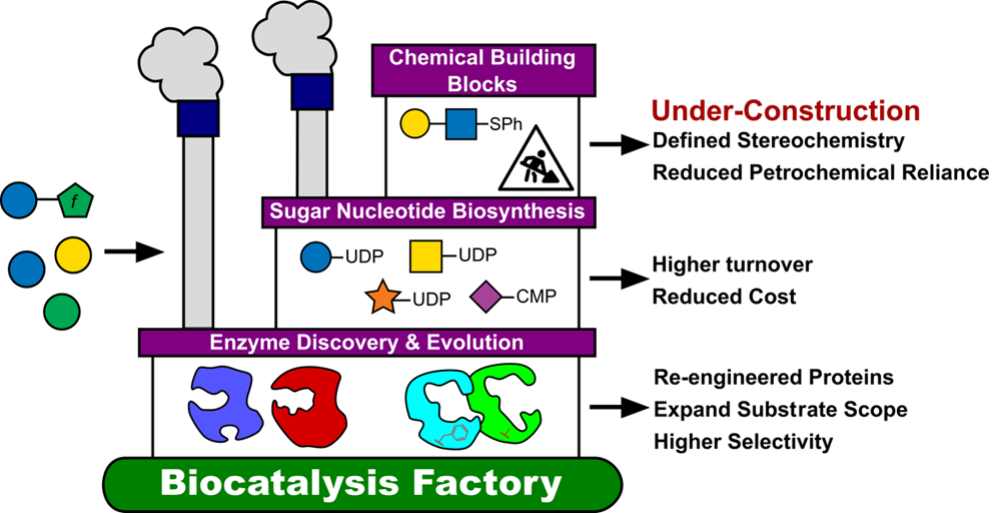

While the field of biocatalysis has bloomed over the past 20–30 years, advances in the understanding and improvement of carbohydrate-active enzymes, in particular, the sugar nucleotides involved in glycan building block biosynthesis, have progressed relatively more slowly. This perspective highlights the need for further insight into substrate promiscuity and the use of biocatalysis fundamentals (rational design, directed evolution, immobilization) to expand substrate scopes toward such carbohydrate building block syntheses and/or to improve enzyme stability, kinetics, or turnover. Further, it explores the growing premise of using biocatalysis to provide simple, cost-effective access to stereochemically defined carbohydrate materials, which can undergo late-stage chemical functionalization or automated glycan synthesis/polymerization.

## 1 Introduction

Glycans are carbohydrate-based biopolymers, linked through individual glycosidic bonds, that are produced by all living organisms. They are an essential group of macromolecules that coat the surface of cells and form an integral part of the intracellular matrix.^1−3^ Many glycans are positioned to modulate or mediate a variety of biological processes, such as signal transduction, host–pathogen recognition, and cellular morphology.^4,5^ Unlike translation and transcription, glycan synthesis is not a templated process which, in addition to the vast range of monosaccharide building blocks available, results in a far greater combinatorial glycan diversity when compared to the macromolecules derived from nucleosides or amino acids.^6^ Further diversity can also be imparted through glycan branching and covalent modification, such as sulfation. As a result of their biological ubiquity, there is a sustained interest from the scientific community around the synthesis of glycan structures. It is here that we focus this perspective, particularly regarding the use of biocatalysis (enzymes) to assemble the building blocks that are required to then enable chemical or biochemical glycan synthesis.

In the case of approaches to biochemical glycan synthesis, sugar nucleotides are the imperative building blocks required. They are composed of an activated sugar donor that is used in glycosylation biosynthetic pathways with a diverse range of acceptors, including glycan chains, releasing an energetic mono- or dinucleotide byproduct.^7,8^ For chemical glycan building block provision, we consider the emerging capabilities of biocatalysis in providing key, staple materials, such as thioglycoside donors and aminosugars. We consider the use of biocatalysis separately within each of these synthesis frameworks, as the challenges surrounding their use differ, but seek to highlight an overall synergy between the use of enzymes and the synthesis of essential glycan feedstock materials.

## 2 Using Biocatalysis to Provide the Building Blocks for Biochemical Glycan Synthesis

Here we discuss recent advances using enzymes to synthesize sugar nucleotides, allowing for more cost- and process-effective access to a broader range of these materials. We consider recent reports (generally during the last five years) and begin with an introduction to mammalian sugar nucleotide biosynthesis to set context, followed by exploring new biocatalysts to assemble and modify the nucleotide diphosphate sugar framework. Moving from in vitro synthesis, we next look at fully enzymatic capabilities, performing consecutive transformations in one pot. Wider discovery aspects surrounding enzyme and bioprocess engineering (e.g., enzyme immobilization) conclude the section.

### 2.1 Mammalian Sugar Nucleotide Biosynthesis: A General Overview

**Figure 1:**
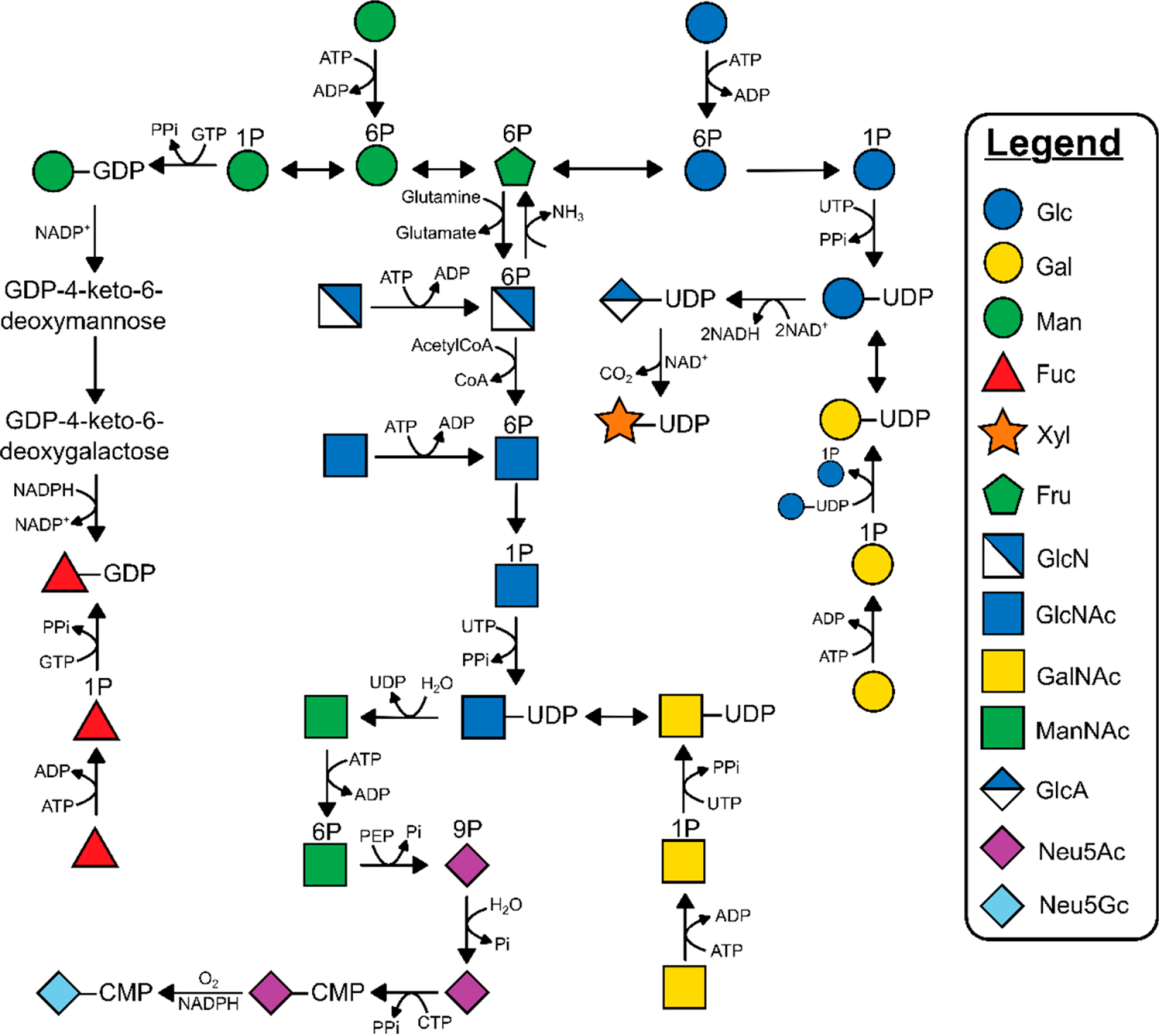
Biosynthesis and interconversion of monosaccharides via de novo and salvage pathways. UDP = uridine diphosphate, CMP = cytidine monophosphate, GDP = guanosine diphosphate.

While there are nine common sugar nucleotides in humans, UDP-d-Glc, UDP-d-GlcNAc, UDP-d-GlcA, UDP-d-Gal, UDP-d-GalNAc, UDP-d-Xyl, GDP-l-Fuc, GDP-d-Man, and CMP-Neu5Ac, more than 50 have been discovered in bacteria, viruses, plants, and other living organisms.^1^ Most recently, the minor nucleotide sugar, UDP-d-Man, was discovered in mammalian cell lines.^9^ The biosynthesis of sugar nucleotides is complicated and achieved via multiple pathways. This includes the interconversion of existing NDP-sugars, salvage pathways, or de novo biosynthesis from free glycans via glycosyl-1-phosphates or the phosphorolysis of glycosidic bonds by glycoside hydrolases such as sucrose phosphorylase (SP) or glucosylglycerate phosphorylase (GGaP).^10−14^Figure 1 includes a snapshot of the de novo synthesis and salvage pathways for mammalian UDP- and GDP-sugar biosynthesis. Notably beyond this, more than 200 intermediates are known to play a role in viral, bacterial, mammalian, and plant NDP-sugar and glycan biosynthesis.^15^

Glucose and fructose are the major carbon sources for organismal monosaccharide biosynthesis.^16−18^ Additional monosaccharides can be salvaged from glycans degraded within lysosomes;^19^ a not insignificant proportion of salvaged amino sugars and sialic acids are transported to the cytosol, reactivated, and reused for de novo glycan synthesis.^20,21^ Before monosaccharides can be used for glycan synthesis, they must be activated to a high-energy donor (most commonly the sugar nucleotide, Figure 1), which act as substrates for Leloir glycosyltransferases. These enzymes utilize activated sugar donors to perform nucleophilic substitution reactions with either retention or inversion of configuration.^22,23^ A significant body of work and detailed reviews focusing on improving the stability, kinetics, and selectivity of glycosyltransferases for synthetic and industrial applications have been published and are thus not considered directly here.^24−27^

The substrate promiscuity for many of the families of enzymes involved in the biosynthesis of sugar nucleotides has not been thoroughly explored. Further, application of biocatalytic approaches (rational design, directed evolution) routinely applied elsewhere to improve substrate scope, kinetics, and/or stability (e.g., for cytochrome P450 and galactose oxidase^28−30^) has only been applied minimally within these families of enzymes. Providing methods to permit cost-effective access to structurally defined glycans via sugar nucleotide building blocks is fundamental to furthering our understanding of their wider roles in regulatory glycobiology.

### 2.2 Discovery of Novel Enzymes to Synthesize Sugar Nucleotides In Vitro

Given the structural diversity of sugar nucleotides, routes for their enzymatic synthesis can become complex and diverse. For most simple NDP-sugars, starting from the hemiacetal, phosphorylation at the anomeric position by kinases, delivers a sugar 1-phosphate. This then undergoes reaction to complete the NDP-sugar, commonly using pyrophosphorylases or uridylyltransferases. From here, further modification can take place, such as epimerization, dehydration, oxidation, and reduction.

Accordingly, enzymes capable of operating at high activity over a pH and temperature range are of great interest due to their simpler integration into one-pot multienzyme (OPME) systems which allow for simplified enzymatic NDP-sugar (and wider glycan) synthesis without the need for tedious purification of anomeric phosphates or NDP-sugars. Additionally, the discovery of enzymes with relaxed substrate specificity permit access to unnatural sugar nucleotides, required for furthering our understanding and control of chemoenzymatic glycan synthesis.

#### 2.2.1 Sugar Kinases

Promiscuous galactokinases (GalKs) such as those from *Steptomyces coelicolor* (*Sc*GalK) and *Leminorella grimontii* (*Lg*GalK) exhibit broad substrate scope, with *Sc*GalK demonstrating activity toward d-Man and l-Ara,^31,32^ an activity previously only observed with human galactokinase (GalK1) mutants, Y379C and Y379W.^31,33^ In contrast to many of the previously characterized bacterial GalKs, *Sc*GalK retains high levels of activity at low temperatures (≈60% activity at 4 °C) and over a broad pH range (>80% of its activity is sustained between pH 4–10).^31,32,34,35^ On the other hand, *Lg*GalK demonstrates substrate acceptance of deoxygalactoses and deoxyfluorogalactoses while retaining high levels of activity outside its optimum temperature.^32^ Unlike *Sc*GalK, *Lg*GalK retains <50% of its activity if the Mg^2+^ cofactor is substituted by other bivalent cations (Ni^2+^, Fe^2+^, Ca^2+^).^32^ These broad tolerances and unusual activity profiles suggest both *Sc*GalK and *Lg*GalK are potential candidates for OPME systems.^36−38^

Outside the immediate discovery of novel enzymes, the development of methodologies for profiling the substrate promiscuity of known wild-type sugar kinases then provides a basis for screening further construct libraries obtained through enzyme evolution. Indeed, ^19^F NMR has provided a basis for screening the promiscuity of 13 wild-type sugar kinases against 17 fluorinated monosaccharides.^39^ More recently, higher-throughput methods utilizing desorption electrospray ionization mass spectrometry (DESI-MS), an ambient ionization technique, have validated these results while providing semiquantitative chemical information and achieving 38 times higher throughput.^40^ The direct infusion of biotransformations to the mass spectrometer (DiBT-MS) and DESI-MS has significant future potential for streamlining the screening of large enzyme libraries (which result from directed evolution or through rational design), permitting more rapid access to enzymes capable of accepting unnatural substrates or which have improved kinetics compared to wild-type.^40−42^

#### 2.2.2 Wild-Type Pyrophosphorylases

Building substrate profiles for UDP-sugar pyrophosphorylases is of interest given the high cost and narrow availability of many common UDP sugars. A novel plant UDP-sugar pyrophosphorylase from *Hordeum vulgare* exhibits favorable biochemical properties including broad pH and temperature tolerances, alongside a wide substrate spectrum (including d-xylose-1-phosphate) and high activity.^43^ Despite having an optimum temperature range of 45–50 °C, only an approximately 20% reduction in activity was observed between 37 and 60 °C,^43^ similar to profiles of UDP-sugar pyrophosphorylases from *Pisum sativum* and *Arabidopsis thaliana*.^44−46^ Activity in the presence of alternative divalent cations (Co^2+^, Ni^2+^, Mn^2+^, and Zn^2+^) alongside this broad pH tolerance makes it amenable to inclusion within multienzyme systems.^43^ Additionally, UDP-sugar pyrophosphorylases from *Arabidopsis thaliana*, *Bifidobacterium infantis*, and *Hordeum vulgare* have demonstrated their effectiveness in simplifying and expanding access to UDP-α-d-xylose and UDP-β-l-arabinose.^43,47^

Building on substantive earlier work to understand plant and bacterial pyrophosphorylase promiscuity,^48−50^ the biocatalytic synthesis of NDP-sugars as targeted inhibitors of their relevant nonmammalian processing enzymes or as polysaccharide chain terminators is a developing area of research.^51−53^ This approach is comparable to alternative chemical pyrophosphorylations, typically requiring prolonged reaction times and often resulting in low yields and complex purifications.^54−56^ In this context, GlmU, a bifunctional *N*-acetylase and uridylyltransferase, has been used for the preparation of C6 and *N*-Ac-modified UDP-d-GlcNAc derivatives in good yields, but this enzyme demonstrates only limited substrate acceptance of C4-pyranose-modified substrates.^52,53,57,58^ Comparatively, a GDP-mannose pyrophosphorylase from *Salmonella enterica* displays good acceptance of C4-modified mannose-1-phosphates but a more limited acceptance for C6 modifications.^59−61^

#### 2.2.3 Sugar Nucleotide Modifying Enzymes

Commercially, UDP-d-Gal and UDP-d-GalNAc are of significantly higher value than their C4 glucose epimers.^62^ As a result, recombinant expression of C4-epimerases which display activity toward UDP-d-Glc and UDP-d-GlcNAc are valuable biocatalysts to reduce cost and improve access to galactosylated NDPs. Uridine diphosphate galactose/glucose C4-epimerases (UGEs) found in plants have recently been extensively reviewed.^63^ However, recently characterized epimerases such as PelX, which displays a preference for *N*-acetylated UDP-hexoses,^64^ and MgUGE, which displays good activity in the presence of Ca^2+^, Co^2+^, Fe^2+^, or Mg^2+^ counterions or EDTA,^65^ provide notable alternatives. The activity of these enzymes under a broad range of conditions allows for their easier integration to multienzyme systems, adding positively to the substantive family of capable C4-epimerases.

The discovery (and expression from *E. coli*) of epimerases which act upon NDP-sugars beyond UDP-sugars is a key interest for industrial potential in rare sugar synthesis and for glycorandomization. In particular, the archaeal epimerase, Gal4E from *Pyrococcus horikoshii* (*Ph*Gal4E_1) displays a preference for GDP-sugars, as well as acceptance of l-sugars, including l-Gal and l-Fuc. Furthermore, RmlC from *S. syringae* accepts dTDP-4-keto-6-deoxy-d-Glc and is a key NDP-sugar for the biosynthetic pathway toward dTDP-l-Rha.^66,67^ Recent work to explore the structure–function relationships in NDP-sugar-active short-chain dehydrogenase/reductase (SDR) enzymes has allowed the identification of patterns of conservation and critical residues, which may serve as a guide for the rational design of SDR enzymes to further expand our capability to access both natural and unnatural NDP-sugars.^68,69^

### 2.3 Fully Enzymatic Syntheses of Sugar Nucleotides

Depending on the buffer, pH, and temperature, sugar phosphates and NDP-sugars can decompose quickly within the time span relevant for enzymatic synthesis. Therefore, their formation in situ from the corresponding reducing sugar has become increasingly popular.^70−72^ Partially driven by their high commercial value, the advent of one-pot multienzyme approaches in the early 1990s has led to simplified access to both natural and a limited range of unnatural NDP-sugars, alongside enabling improved characterization of glycosyltransferases. This section therefore focuses only on recent advancements in one-pot, biosynthetic routes to NDP-sugars, particularly around the regeneration of expensive cofactors, which are often required stoichiometrically.

#### 2.3.1 OPME Synthesis of NDP-Sugars

**Scheme 1:**
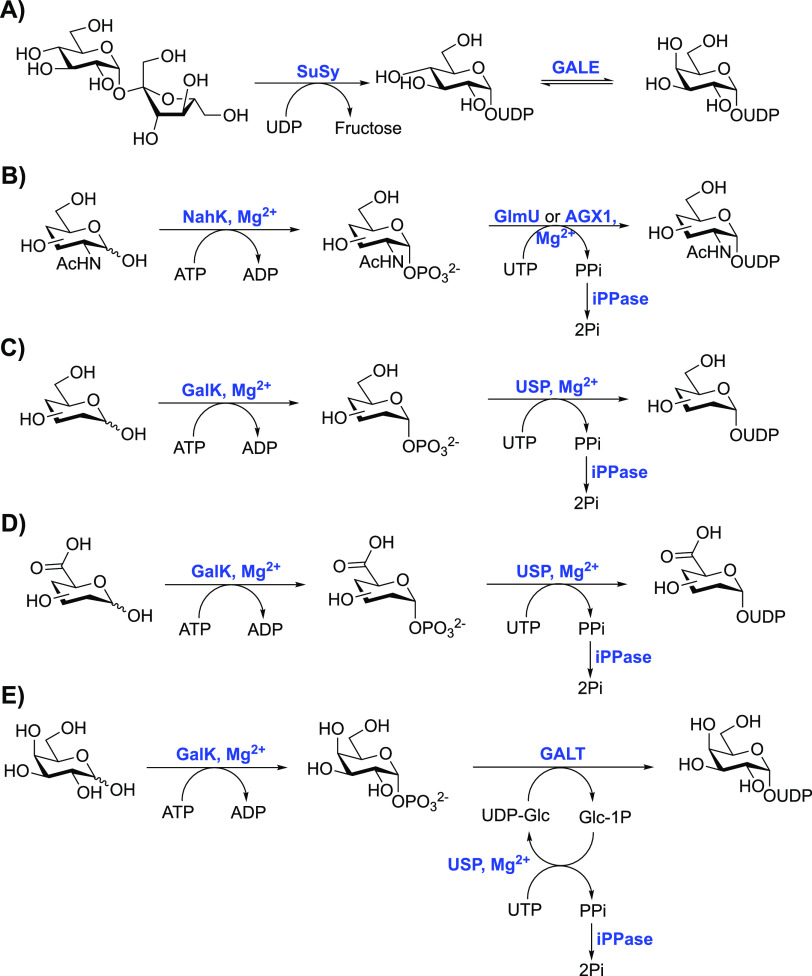
Selection of Methods Used to Access UDP/GDP-Sugars in One Pot from Mono- or Disaccharides

Scheme 1 highlights recent methods utilized to prepare NDP-sugars in situ for subsequent use with glycosyltransferases. This includes sucrose synthase (SuSy), which provides UDP-d-Glc to then undergo C4-epimerization and provide cheap access to UDP-d-Gal (Scheme 1A).^73^ The use of galactokinase (GalK) or *N*-acetylhexosamine 1-kinase (NahK) has provided the corresponding anomeric phosphates, followed by pyrophosphorylative coupling using UDP-pyrophosphorylase (USP) or GlcNAc-1P uridylyltransferase (GlmU/AGX1) to grant UDP-hexose/hexosamine or uronate sugars (Scheme 1B–E).^74−76^ Due to the reversible nature of this pyrophosphate formation, the inclusion of inorganic pyrophosphatase (iPPase) hydrolyzes the inorganic pyrophosphate produced (to inorganic phosphate), thus rendering the coupling reaction irreversible. The inclusion of galactose-1-phosphate uridylyltransferase (GALT) following phosphorylation has enabled easier access to unnatural UDP-d-Gal derivatives, where a suitable UDP-pyrophosphorylase (USP) cannot be found (Scheme 1E).^77,78^ Through the binding of UDP-d-Glc to the catalytic histidine residue of GALT, glucose-1-phosphate is released and subsequently replaced by the galactose-1-phosphate derivative; the displaced glucose-1-phosphate is in turn used to regenerate UDP-d-Glc using UDP-pyrophosphorylase.^79^ This methodology has been used with success to generate nucleotide diphosphate derivatives of monodeoxygalactoses, monodeoxyfluorogalactoses, and galactosamine, using only a catalytic quantity of UDP-glucose,^77^ and has also enabled the preparation of nucleobase-modified UDP-sugars.^78^ Finally, GTP-l-fucose derivatives can be produced in situ from l-fucose using a bifunctional l-fucokinase/GDP-fucose pyrophosphorylase (FKP) isolated from *Bacteroides fragilis* (Scheme 1F).^74−76,80^

**Figure 2:**
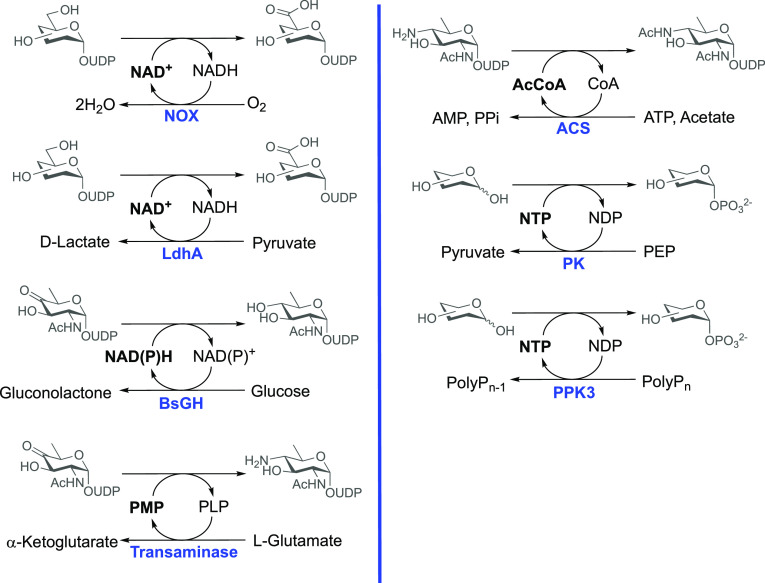
Common systems used for regeneration of cofactors and nucleotide triphosphates used for sugar nucleotide biosynthesis. Enzymes: NOX, NADH oxidase; LdhA, lactate dehydrogenase; BsGH, d-glucose dehydrogenase; ACS, acetyl-CoA synthase; PK, pyruvate kinase; PPK3, polyphosphate kinase; PEP, phosphoenolpyruvic acid.

Generally, the poor availability and high cost of NDP-sugars are considered bottlenecks for Leloir glycosyltransferase-mediated glycan synthesis. Recently, it was reported that such cost-effectiveness (and hence viability for multienzyme cascades) had been further improved through regeneration of the required cofactors (NADH, NADPH, NAD^+^, PMP, and acetyl-CoA, Figure 2).^81,82^ This has recently been exemplified through the synthesis of 25 challenging to access NDP-sugars starting from mannose, sucrose, and *N*-acetylglucosamine, in high yield, on a multigram scale, and without the need for purifications.^81,82^ Additionally, the use of polyphosphate kinase (PPK3) or pyruvate kinase (PK) allows for the regeneration of the nucleotide triphosphates using phosphate glass or phosphoenolpyruvic acid (PEP) as sources of phosphate (Figure 2).^83−87^ Both methods were effective at regenerating ATP used by sugar kinases and UTP/GTP utilized by NDP-sugar pyrophosphorylases.

#### 2.3.2 OPME Synthesis of NMP-Sugars

**Scheme 2:**
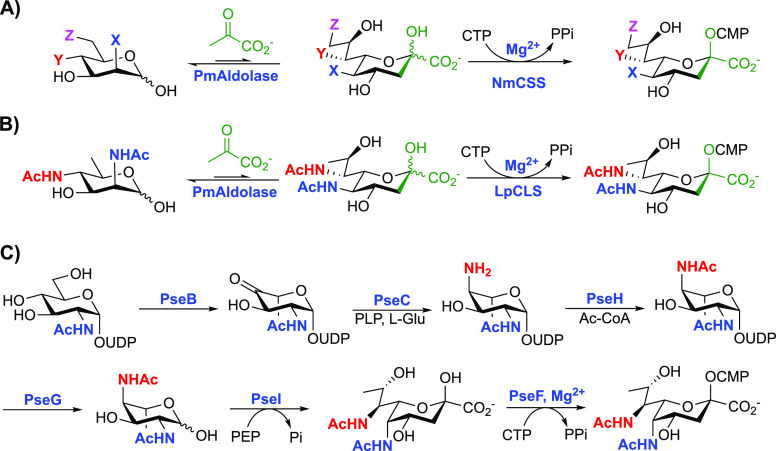
(A) OPME synthesis of CMP-Neu5Ac derivatives from ManNAc derivatives using *P. multocida* sialic acid aldolase (*Pm*Aldolase) and *N. meningitidis* CMP-sialic acid synthetase (*Nm*CSS). X = NHAc, N_3_, NHGc, NHAcN_3_, NHAcF, or NHDAz; Y = NHAc, N_3_, F, or OH; Z = OH, NHAc, or N_3_. (B) OPME synthesis of CMP-Leg5Ac7Ac from 6-deoxy Man2Ac6Ac using *P. multocida* sialic acid aldolase (*Pm*Aldolase) and *L. pneumophila* CMP-5,7-di-*N*-acetyllegionaminic acid synthetase (*Lp*CLS). (C) Biosynthetic pathway to CMP-Pse5Ac7Ac from UDP-GlcNAc using PseB (UDP-GlcNAc 4,6-dehydratase), PseC (UDP-4-amino-4,6-dideoxy-*N*-acetyl-β-l-altrosamine transaminase), PseH (UDP-4-amino-4,6-dideoxy-*N*-acetyl-β-l-altrosamine-*N*-acetyl transferase), PseG (UDP-2,4-diacetamido-2,4,6-trideoxy-β-l-altropyranose hydrolase), PseI (pseudaminic acid synthase) and PseF (pseudaminic acid cytidylyltransferase) from *A. cavaie* and *C. jejuni*.^99,100^

Nonulosonic acids are a diverse family of nine-carbon monosaccharides commonly found as terminal residues in glycans presented as cell surface glycoproteins/glycolipids. Indeed, more than 50 structurally distinct forms of sialic acid have been found in nature;^88,89^ the development of methods to facilitate their cost-effective and facile introduction into glycans is therefore of paramount interest. Due to the chemical lability of NMP-sugars such as CMP-Kdo (CMP-ketodeoxyoctonic acid),^90,91^ OPME sialyation systems have been developed to access sialylated glycans. To illustrate, the sialic acid aldolase from *Pasteurella multocida* (*Pm*Aldolase) has demonstrated a wide substrate acceptance, allowing for controlled access to modified CMP-sugars starting from derivatives of mannose (Scheme 2A).^92−96^ This system could be coupled with CMP-sialic acid synthetase from *Neisseria meningitidis* (*Nm*CSS) and a range of sialic acid transferases (*Pm*ST1, *Pm*ST3, *Pd*2,6ST, *Cj*CstII, *Nm*SiaD_w_) to incorporate the CMP-sugar into glycans (with varying levels of success).^92−95^ For neuraminic acid, this system has proven effective for synthesizing unnatural derivatives, incorporating azide,^92−95^ diazirine,^94^ and fluorine.^96^ The ability to efficiently synthesize and incorporate both diazirinated and fluorinated glycans is key to enabling access to chemical biology probes for photo-cross-linking experiments.

The bacterial nonulosonic acids, legionaminic acid (Leg) and pseudaminic acid (Pse), remain particularly underexplored compared to neuraminic acid (Neu5Ac).^97^ Beyond derivatives of Neu5Ac, the use of *Pm*Aldolase in combination with *L. pneumophila* CMP-5,7-di-*N*-acetyllegionaminic acid synthetase (*Lp*CLS) has provided an efficient route to access CMP-Leg5Ac7Ac from 6-deoxy mannosamines (Scheme 2B).^98^ Additionally, the biosynthesis of CMP-Pse5Ac7Ac was recently reported on a milligram scale using enzymes from *Campylobacter jejuni* (PseBCHGI) and *Aeromonas caviae* (PseF), providing a practical route to CMP-Pse5Ac7Ac in vitro from UDP-d-GlcNAc (Scheme 2C).^99,100^ Using optimized in vitro conditions, PseB,C,H,G,I could be used in “one pot”, permitting access to Pse5Ac7Ac. Performing the reactions of PseB and C together is vital as in addition to PseB acting as 5-inverting 4,6-dehydratase it also catalyzes the C5-epimerization of UDP-4-keto-6-deoxy-β-l-IdoNAc to UDP-4-keto-6-deoxy GlcNAc. Together, these routes have the potential to provide increased access to under explored CMP-sugars, and some mammalian and bacterial sialyltransferases display promiscuity to utilize CMP-Leg5Ac7Ac, such as porcine ST3Gal1 and human ST6Gal1.^101^

### 2.4 Bioprocess and Enzyme Engineering Strategies for Sugar Nucleotide Synthesis

#### 2.4.1 Enzyme Engineering

Enzyme engineering has become a central tool for biocatalytic approaches to the synthesis of fine chemicals.^102^ However, engineering to further the synthesis of sugar nucleotide precursors, the donors themselves, or to access carbohydrate building blocks for chemical synthesis is, comparably, still in its infancy. Notwithstanding this, the pioneering work of Thorson (rationally designed galactokinases) and others sets an exciting platform for this to develop.^103−105^ This section will discuss some recent examples and consider the application and technology advancement prospects for developing improved enzyme variants with altered substrate acceptance and expanded promiscuity for use in carbohydrate building block synthesis.

##### 2.4.1.1 In Vivo Synthesis with Engineered Enzymes

**Figure 3:**
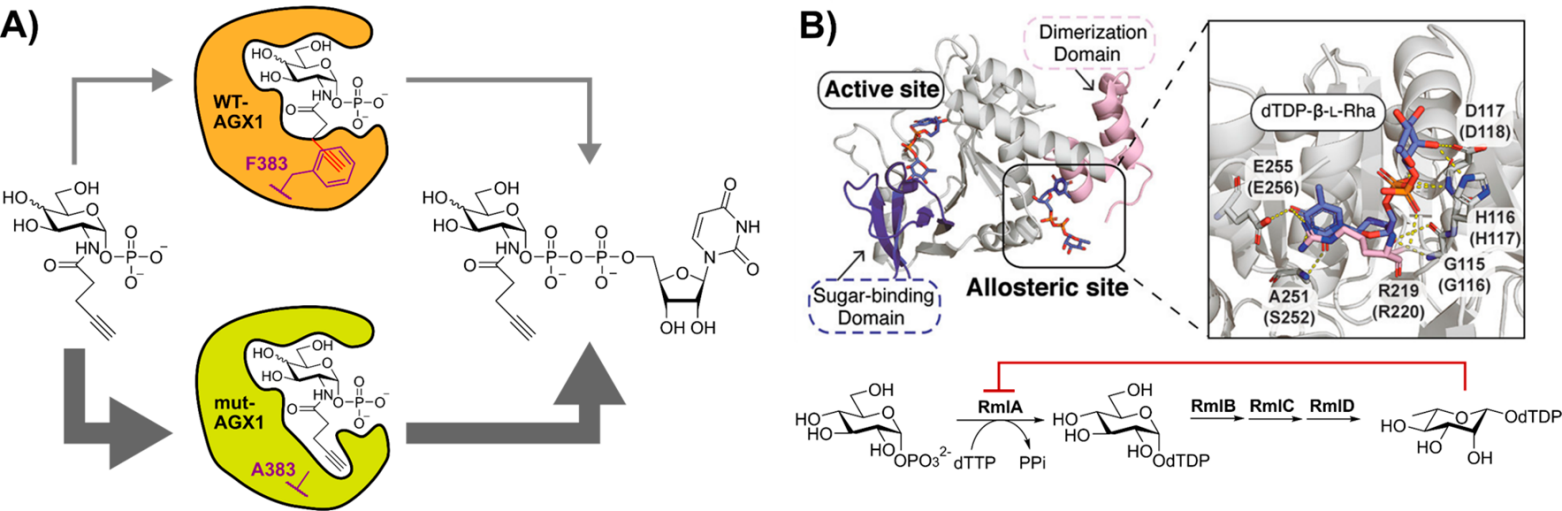
(A) Biosynthesis of UDP-d-GlcNAlk and UDP-d-GalNAlk in vitro using a mutant AGX1 modified to introduce a “hole” capable of allowing acceptance of alkyne modification. Adapted and reproduced from ref (108) under a CC-BY 4.0 license. Copyright 2021 American Chemical Society. (B) Structure of Gram-negative nucleotidyltransferase RmlA (PDB ID: 1G3L). Catalytic activity is regulated through binding of dTDP-l-Rha to the allosteric site (enlarged, residue labeled for *P. aeruginosa* RmlA and homologous *Salmonella enterica* LT2 (in parentheses)). Modification to the allosteric site permits enhanced activity and expanded substrate scope. Adapted and reproduced from ref (111). Copyright 2022 American Chemical Society.

While not strictly used to make discrete building blocks for glycan assembly, multiple biosynthetic pathways containing engineered enzymes have been demonstrated which incorporate modified sugar building blocks, including NDP-sugars.^106,107^ Using a “bump-and-hole” approach, proteins can be glycosylated with non-natural sugars using an artificial biosynthetic pathway.^108,109^ Through mutation, a protein cavity can be introduced, providing the space required for unnatural functional groups to be accepted. Accordingly, the engineering of AGX1 [mut-AGX1, AGX1(F383A)] and GalNAc-T [BH-GalNAc-T, GalNAc-T(I253A/L310A)] enabled a demonstration that alkyne-modified d-GalNAc derivatives could be selectively installed across the glycoproteome via the UDP-sugar (Figure 3A).^110^ The substrates that were synthesized in this manner contained butynyl or pentynyl appendages to the *N*-acetyl carbon, which were accommodated by the expanded enzyme pocket. If these methods could be harnessed in vitro, they could lead to accessing new panels of modified UDP-sugars.

##### 2.4.1.2 In Vitro Synthesis with Engineered Enzymes

As an alternative to bump-and-hole modifications of the active site, rational mutation of the allosteric site of the nucleotidyltransferase RmlA was recently demonstrated, leading to expanded substrate tolerance and improvements in catalytic activity.^111^ RmlA is responsible for catalyzing the formation of dTDP-d-Glc from Glc-1-phosphate and deoxythymidine triphosphate in vivo. The catalytic efficiency of RmlA is limited by a negative feedback inhibition loop, through binding of dTDP-l-Rha to the allosteric site (Figure 3B).^112^ Mutation of the allosteric site resulted in subtle changes in the protein quaternary secondary structure and reduced inhibition, permitting improved catalytic efficiency and enhanced access to dTDP-d-Man, dTDP-d-Gal, dTDP-d-GlcNAc, and dTDP-d-Fuc.

##### 2.4.1.3 Technology and Methodology for Engineered Enzyme Library Screening

The detection and characterization of biotransformations is an important aspect in the development of new biocatalytic methods to access glycan building blocks. In this regard, a simple phenol-based detection method toward nucleotide sugar 4,6-dehydratases (NSDs) was recently developed, which enabled high-throughput analysis using crude enzymes for the first time.^113^ Using resorcinol, a phenolic compound which reacts with the ketone within the oxidized product of NSD action, absorbance could be monitored at 510 nm. Compared with previous methods, which monitored product formation at 320 nm, less background interference was observed. While simple, this allowed cell-free extract (CFE) to be used instead of purified enzyme, which significantly reduced the time associated with reaction screening. The quantitative nature of observed absorbance permitted kinetic characterization of a panel of mutants against dTDP-l-Glc. High-throughput screening methodology is an essential technology to generate large volumes of data. Recent advances in data analysis and using artificial intelligence to analyze large data sets means methods like those described above are an important step toward the improvement of enzymatic transformations and reaction outcomes. Incorporation of inline data analysis and modeling software seems a logical next step toward automating mutant quantification and reaction characterization.

#### 2.4.2 Bioprocess Engineering

Concomitantly to discovering and engineering enzymes, the design and optimization of effective bioprocesses to deliver scalability and industrial relevance is vital. The cost of production of recombinant proteins can often mean increased economic efficiency is accessible if enzymes are immobilized onto solid supports. This enables simpler recovery and reuse of the enzyme and increases overall productivity, through increasing the total turnover number, while reducing overall cost through reuse. Immobilization can also permit transfer into a continuous process.^114^ This section discusses technological advances that have improved biocatalytic NDP-sugar synthesis.

##### 2.4.2.1 OPME Systems for NDP-Sugar Synthesis

**Figure 4:**
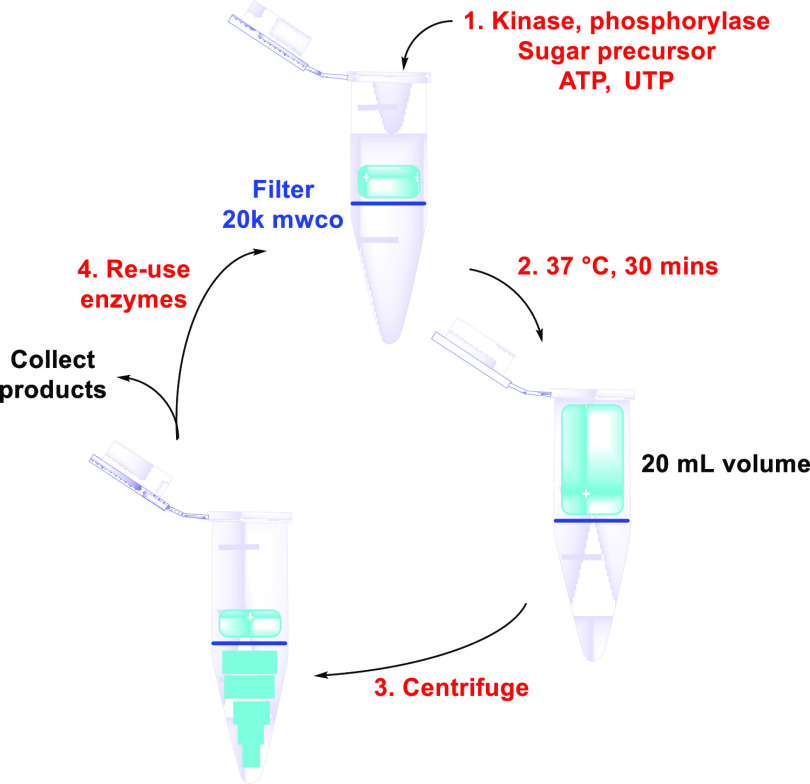
Repetitive batch mode synthesis of NDP-sugars using a centrifugal filter. MWCO, molecular weight cutoff.

The enzymes used to produce UDP-d-Gal, UDP-d-GalNAc, and UDP-d-GlcNAc can now be used in a repetitive batch mode synthesis without the need for extensive equipment,^115^ reducing the requirement for costly and laborious large-scale protein production and purification. These NDP-sugars can be prepared in multigram quantities with the enzymes involved used repetitively for up to 24 cycles, equating to a high mass-based total turnover number of 398–522 g_product_/g_enzyme_. This was achieved using a centrifugal filter (Figure 4). The reaction mixture was made up in tube, run for 30 min, and centrifugal filtration then separated the completed reaction from the biocatalysts with a 20,000 kDa cutoff. New reaction constituents were added to the recycled enzymes and the cycle repeated. More recently, this technology has been applied to the preparation of GDP-l-Fuc, achieving a high total turnover number of 31 g_product_/g_enzyme_ after 15 cycles.^116^ While this would be prohibitively expensive to be applied on scale due to the cost of centrifugation and the spin filters, it demonstrates an important principle surrounding the reuse of the enzymes. Continuous filtration devices are readily available in more economic forms, so this could be applied in a more cost-effective system. The key here is the demonstration of biocatalyst longevity from consideration of a stability to activity axis.

**Figure 5:**
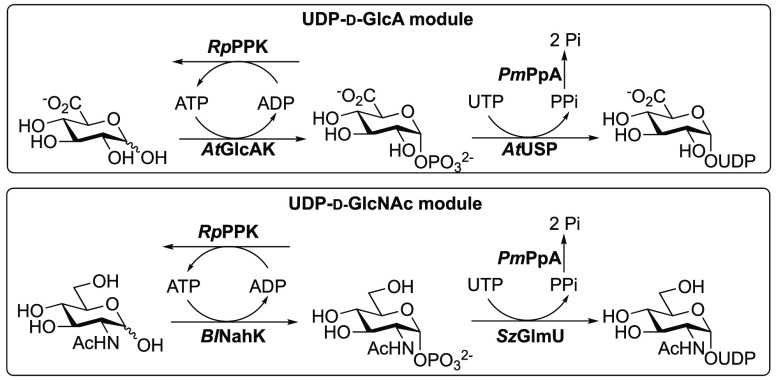
Module enzyme assemblies for the production of UDP-d-GlcA and UDP-d-GlcNAc.

Several studies that detail different aspects of one-pot hyaluronic acid (HA) synthesis have been reported recently. Individual enzyme modules could be used to produce UDP-d-GlcA and UDP-d-GlcNAc building blocks (Figure 5), which were then fed into a hyaluronan module for HA synthesis using pmHAS.^117^ UDP-d-GlcA was synthesized from d-GlcA, using a GlcA kinase (*At*GlcAK) and UDP-sugar phosphorylase (*At*USP), both from *Arabidopsis thaliana*. The UDP-d-GlcNAc was synthesized in a similar manner using GlcNAc 1-phosphate kinase (*Bl*NahK) from *Bifidobacterium longum* and UDP-d-GlcNAc pyrophosphorylases from *Streptococcus zooepidemicus* (*Sz*GlmU) or *Campylobacter jejuni* (*Cj*GlmU). Both units were also paired with a pyrophosphatase from *P. multocida* (*Pm*PpA) to hydrolyze the PPi. This approach was different from an earlier report which used sucrose as the starting material for UDP-d-GlcA and employed UDP-d-Glc dehydrogenase (UGDH) to oxidize UDP-d-Glc.^118^ This required the use of NAD^+^, so the contemporary report avoided the need for the NADH oxidase (NOX) system for NAD^+^ recycling. UDP-d-GlcA was produced in 84% yield (8.4 mM) after 1 h. This equated to a space–time yield of 16 mmol L^–1^ h^–1^ but was only on a 300 μL scale. Similarly, UDP-d-GlcNAc could be synthesized continuously to 9.8 mM (98% yield), but once again only at 300 μL. Additionally, the modules illustrated in Figure 5 could be improved through the addition of polyphosphate kinase from *Ruegeria pomeroyi* (*Rp*PPK) to permit ATP recycling,^119^

Scale must be properly addressed to ensure valuable NDP-sugar building blocks can be obtained in adequate quantities. Developments such as those above demonstrate that there are means to achieving this and provide a glimpse of what is possible and where resources should be focused to ensure bioprocess development is not forgotten.

##### 2.4.2.2 Immobilization of Sugar-Nucleotide Producing Enzymes

Recently,^119^ the entire cascade illustrated in Figure 5 (including *Pm*HAS for HA synthesis) was immobilized onto magnetically recoverable beads, and the system reused up to five times.^120^ These modules have also been compartmentalized and transferred into continuous flow reactors to permit automated glycan (HA) synthesis,^121^ although no information with regard to scale was disclosed in this most recent report.

The synthesis of UDP-d-Glc using immobilized pyrophosphorylase from *Thermocrispum agreste* (*Ta*GalU) was also reported recently.^122^ This thermostable enzyme was active at 50 °C for 30 min, losing up to 65% of its activity over this time. However, *Ta*GalU immobilized on amino-functionalized mesostructured cellular foams (MCFs) demonstrated retained activity up to 80 °C (40% activity after 5 h) and for greater lengths of time (up to 96 h). These reactions were carried out on a preparative (10 mL) scale at 2 mM substrate concentration, and the immobilized enzyme could be reused up to five times.^122^

**Figure 6:**
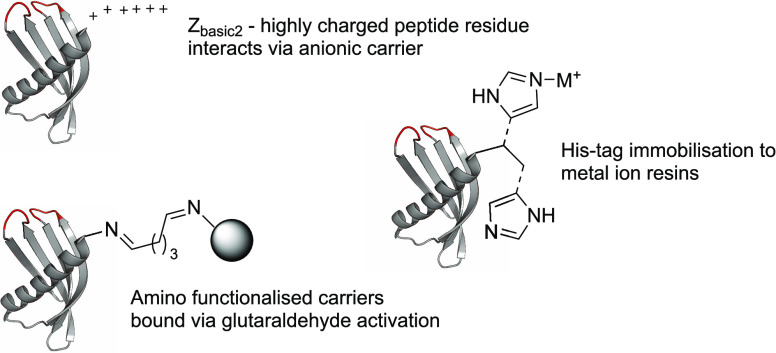
Common immobilization methods frequently used, including peptide affinity (Z_basic2_), charge affinity via His-tag, and covalent binding/glutaraldehyde activation.

The immobilization of three enzymes required for the synthesis of CMP-Neu5Ac within hydrogels as a method to avoid CTP cross-inhibition of *N*-acyl-d-glucosamine 2-epimerase (AGE) and *N*-acetylneuraminate lyase (NAL) has been described.^123^ Utilizing a confinement and compartmentalization strategy enabled higher substrate concentrations to be used. Immobilization has also been used to improve the overall efficiency of sialyl galactoside synthesis using a two-step route from Neu5Ac.^124^ Initially, immobilization via N-terminus His-tags of CMP-sialic acid synthetase from *Neisseria meningitis* (*Nm*CSS) and sialyltransferase from *Pasteurella dagmatis* (*Pd*SiaT) yielded poor immobilizates in all instances (via affinity or epoxy on a range of commercial carriers). Therefore, constructs containing the Z_basic2_ protein module (a 7 kDa, 58 amino acid module with a high charge ratio) were made and tested, affording high immobilization yields and full activity recovery on all of the carriers versus the native enzymes (Figure 6). These constructs could also be selectively immobilized from the crude cell extracts. The immobilized enzymes were used to synthesize α-2,3-Neu5Ac-4NP-β-d-Gal over five cycles, maintaining a yield of >75% every time. This was only performed on an analytical scale but demonstrates potential for long-term (repeated) use of the enzymes, transfer to continuous reactors, and the immobilized preparation of unnatural CMP-Sia derivatives as described in section 2.3.2.

**Figure 7:**
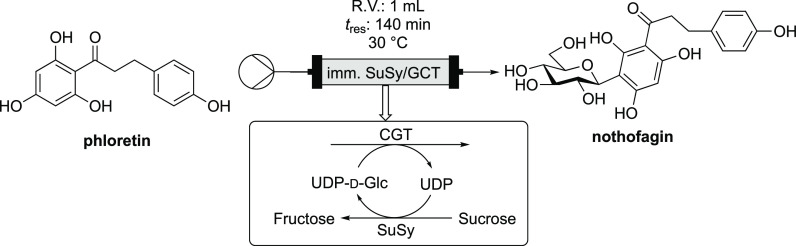
Continuous flow module using immobilized SuSy and GCT for UDP-d-Glc production and applied in nothofagin synthesis.

Sucrose synthase (*Gm*SuSy) could also be immobilized on ReliSorb via the Z_basic2_ module for continuous production of UDP-d-Glc and also utilized in a two-enzyme continuous packed-bed reactor for a flow synthesis of the flavonoid nothofagin (Figure 7).^125,126^ This built on earlier work showing that UDP-d-Glc could be produced reliably with SuSy used in whole cell form, generating total turnover numbers in the region of 70–230 g_product_/g_cells_. Impressively, this was scaled up to produce UDP-d-Glc on a 41 g scale, with a specially adapted processing procedure affording 31 g in a 63% isolated yield.^127^ As with the batch work, catalytic quantities of UDP could be used, and the continuous reactor maintained at least 50% conversion for up to 90 reactor cycles (140 min residence time, Figure 7).

The scalability of this was demonstrated in combination with a glycosyltransferase from rice (*Os*GCT) which used UDP-d-Glc to glycosylate a phenol and produce nothofagin, a C-linked, glycosylated flavonoid.^128^ This one-pot, two-step process was shown to work on 50 g scale, with a 98% isolated yield of nothofagin.

Immobilization has also been harnessed to replicate a biosynthetic pathway in vitro.^129^ A seven-enzyme biosynthetic pathway was proposed for the synthesis of the l-rhodosamine-thymidine diphosphate (TDP), but when expressed in *E. coli*, several of the biocatalysts were insoluble and precipitated during the reaction. Consequently, all of the enzymes were immobilized via His-tags to TALON affinity beads and run in a OPME cascade reaction.

The immobilization of carbohydrate-active biocatalysts offers a multitude of benefits. The challenge for bioprocess scientists moving forward is being able to rationalize immobilization more accurately, as current methods do not generally permit rapid carrier selection or immobilization process optimization. Recent exciting work has though developed a flow system with inline analysis for a “data-rich” optimization method of enzyme immobilization.^130^ This interesting approach could reduce the labor associated with enzyme immobilization and be adopted and applied more broadly within NDP-sugar synthesis approaches.

## 3 Using Biocatalysis to Provide the Building Blocks for Chemical Glycan Synthesis

The biocatalytic synthesis of building blocks for use in chemical synthesis of glycan targets is a burgeoning area of interest; in particular, this is driven by a desire to access stereochemically defined materials in the absence of extensive protecting group strategies.

### 3.1 Thioglycosides

**Figure 8:**
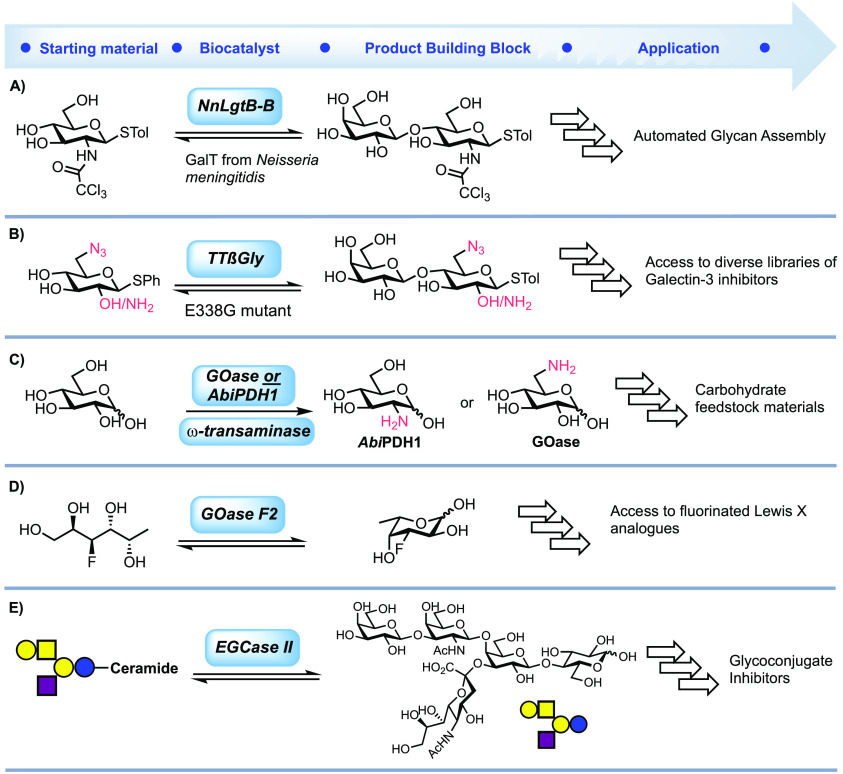
Summary of biocatalytic methods used to deliver building blocks appropriate for chemical glycan synthesis.

1-Thioglycosides scarcely occur in Nature, with most members belonging to glucosinolates; however, they are routinely utilized as glycosyl donors for synthetic carbohydrate chemistry.^131^ Recently, biocatalysis was utilized to provide robust and simple access to a LacNAc thioglycoside building block for automated glycan assembly (AGA, Figure 8A).^132^ First, *Nm*LgtB-B was shown to use lactose as an inexpensive donor ofd-Gal for thiodisaccharide synthesis. Additionally, a one-pot three-enzyme cycle whereby UDP-d-Gal was produced in situ using sucrose synthase in combination with Glc_(1,4)_-epimerase was investigated. Both methods were successful, but the former was preferred for generating usable hundred milligram quantities of thioglycoside building blocks, thereafter only requiring acetyl protection of the hydroxyl groups, before being suitable for AGA. The latter catalytic cycle did though avoid the use of high concentrations of UDP or lactose, which may not be compatible with other glycosyltransferases.

Glycosynthases obtained through mutagenesis of glycosyl hydrolases (removing/reducing hydrolysis activity) are an attractive alternative to glycosyltransferases as they do not require expensive ND(M)P-sugar donors.^133−136^ The E338G mutant of the *Thermus thermophilus* glycoside hydrolase (*TT*βGly E338G) was successfully used in combination with a glycosyl fluoride donor and C6-modified acceptor for the expeditious synthesis of lactose and lactosamine core thioglycosides (Figure 8B).^137,138^ Additionally, β-d-glucuronidase *Dt*GlcA from *Dictyoglomus thermophilum* has shown promise as a future thioglycoligase capable of catalyzing the formation of *S*-glucuronides.^139^ Mutation of the catalytic E396 residue to glutamine led to an efficient catalyst for synthesizing a small panel of glucuronic acid thioglycosides and lays a foundation for the synthesis of *S*-glycoside building blocks.^140^

### 3.2 Amine- and Fluorine-Containing Sugars

Amino-functionalized sugars have important roles, for example, within therapeutic carbohydrates, and a recent report demonstrated a two-enzyme cascade could be employed to afford 2- and 6-amino galactose units (Figure 8C).^141^ The cascades proceeded with oxidation of the 2- or 6-OH positions with pyranose dehydrogenase from *Agaricus bisporus* (*Abi*PDH1) or galactose oxidase from *Fuasrium graminearum* (*Fgr*GOase), respectively. These were then combined in a subsequent reaction with a transaminase to afford the aminosugar products. An engineered galactose oxidase (GOase) was also used in the synthesis of 3-deoxy-3-fluoro-l-fucose from the fluorinated fucitol (Figure 8D).^142^ The engineered GOase F_2_ variant showed the highest activity, and the reaction was successfully scaled up to a 2.1 mmol scale and proceeded in a 59% isolated yield.

### 3.3 Ganglioside-Derived Glycans

Endoglycoceramidases (EGCases) catalyze the hydrolysis of the glycosidic linkage between the oligosaccharide and ceramide of various glycosphingolipids; their substrate specificity and use as both ceramidases and glycosynthases is a growing area of interest.^143^ The novel EGCase I from *Rhodococcus equi* 103S (103S_EGCase I) has been demonstrated to express at a high level (80 mg L^–1^ media) from *E. coli* and with specific activities between 0.45 and 6.44 nmol/min/μg enzyme for a range of substrates (Gb4-Cer, Lac-Cer, GM3, GM1, and fucosyl-GM1).^144^ More recently, EGCase II from *Rhodococcus* sp. was used to enzymatically hydrolyze GM1 ganglioside (Figure 8E). The resulting GM1 oligosaccharide was functionalized at the anomeric center via two separate methods: oxime ligation and DMC-mediated glycosyl azide formation.^145^ EGCases have the potential to be potent biocatalysts to provide access to complex glycan starting materials (for further synthesis) from biological sources, without the need for lengthy enzymatic synthesis. However, their use more widely has been hampered by typically challenging/poor expression and stability.^144,146^

## 4 Outlook

Delivering building blocks for glycan synthesis using enzymes still rests largely on the provision of sugar nucleotides. Significant advancements have been made (especially cofactor recycling for in situ approaches). This is coupled with continued exploration to discover promiscuous enzymes that can build non-native NDP-sugars, especially as care must be taken in quantifying unnatural glycan processing.^147^ Only recently has substrate scope for glycosyl kinases started to be routinely investigated as part of their initial characterization. As a result, access to glycosyl phosphates is not cost-effective for many research groups and this percolates, such that exploring substrate scope becomes a challenging and unattractive part of characterizing novel pyrophosphorylases and/or nucleotidyltransferases.

Exploring process engineering and effective use/recycling of stable/functional enzymes for NDP-sugar provision is an important direction of travel, alongside consideration that modification of carbohydrate-active enzymes should be beyond just active sites. Computational design will continue to play an important role. This has recently allowed for the expression of human heparanase (a glucuronidase) from *E. coli* with identical kinetics, inhibition, structure, and structural/protein dynamics to the wild-type protein.^148^ The use of the PROSS-2 server (the protein repair one-stop shop) to improve the expression levels/stability of known enzymes by incorporating evolutionally conserved and functionally neutral mutations could be a game changer in optimizing proteins which exhibit favorable expression levels and stability to make many of these enzymes attractive to industrial applications.^149^ Finally, for the provision of glycan building blocks for new technologies (such as AGA) or traditional chemical building blocks for synthesis, one of the defining bottlenecks is scalable access to such materials; biocatalytic approaches are showing they can innovate here.

Overall, a unifying method or technology for NDP-sugar synthesis, be that enzymatic, chemoenzymatic, or chemical, remains elusive. This may well indeed be an unrealistic expectation, but the positive viewpoint is that the harmony of biocatalysis with glycan building block synthesis looks set to continue, much as it has been enabling for the production of other fundamental biological building blocks.^150^
